# Tracing vitamins on the long non-coding lane of the transcriptome: vitamin regulation of LncRNAs

**DOI:** 10.1186/s12263-024-00739-4

**Published:** 2024-03-12

**Authors:** Fatemeh Yazarlou, Fatemeh Alizadeh, Leonard Lipovich, Roberta Giordo, Soudeh Ghafouri-Fard

**Affiliations:** 1 Center for Childhood Cancer, Abigail Wexner Research Institute at Nationwide Children’s Hospital, Columbus, OH USA; 2 College of Medicine, Mohammed Bin Rashid University of Medicine and Health Sciences, Box 505055, Dubai, United Arab Emirates; 3 Department of Genomic Psychiatry and Behavioral Genomics (DGPBG), Roozbeh Hospital, School of Medicine, Tehran University of Medical Sciences, Tehran, Iran; 4 Department of Biology, College of Science, Mathematics, and Technology, Wenzhou-Kean University, Wenzhou, Zhejiang Province China; 5 Shenzhen Huayuan Biological Science Research Institute, Shenzhen Huayuan Biotechnology Co. Ltd., 601 Building C1, Guangming Science Park, Fenghuang Street, 518000 Shenzhen, Guangdong People’s Republic of China; 6 Center for Molecular Medicine and Genetics, School of Medicine, Wayne State University, 3222 Scott Hall, 540 E. Canfield St., Detroit, MI 48201 USA; 7 Department of Biomedical Sciences, University of Sassari, Viale San Pietro, Sassari, 07100 Italy; 8 Department of Medical Genetics, Shahid Beheshti University of Medical Sciences, Tehran, Iran

**Keywords:** Vitamins, Long non-coding RNA (lncRNA), Noncoding RNA, Nutrition, Nutrigenomics, Functional foods

## Abstract

A major revelation of genome-scale biological studies in the post-genomic era has been that two-thirds of human genes do not encode proteins. The majority of non-coding RNA transcripts in humans are long non-coding RNA (lncRNA) molecules, non-protein-coding regulatory transcripts with sizes greater than 500 nucleotides. LncRNAs are involved in nearly every aspect of cellular physiology, playing fundamental regulatory roles both in normal cells and in disease. As result, they are functionally linked to multiple human diseases, from cancer to autoimmune, inflammatory, and neurological disorders. Numerous human conditions and diseases stem from gene-environment interactions; in this regard, a wealth of reports demonstrate that the intake of specific and essential nutrients, including vitamins, shapes our transcriptome, with corresponding impacts on health. Vitamins command a vast array of biological activities, acting as coenzymes, antioxidants, hormones, and regulating cellular proliferation and coagulation. Emerging evidence suggests that vitamins and lncRNAs are interconnected through several regulatory axes. This type of interaction is expected, since lncRNA has been implicated in sensing the environment in eukaryotes, conceptually similar to riboswitches and other RNAs that act as molecular sensors in prokaryotes. In this review, we summarize the peer-reviewed literature to date that has reported specific functional linkages between vitamins and lncRNAs, with an emphasis on mammalian models and humans, while providing a brief overview of the source, metabolism, and function of the vitamins most frequently investigated within the context of lncRNA molecular mechanisms, and discussing the published research findings that document specific connections between vitamins and lncRNAs.

## Introduction

Genome-scale biological studies in the post-genomic era have revealed an unprecedented level of complexity in the genome, spurring the ever-growing expansion of new frontiers in genomics-driven and precision medicine. A direct outcome of the efforts of international consortia, such as Functional Annotation of the Mammalian Genome (FANTOM) and Encyclopedia of DNA Elements (ENCODE), in the first post-genomic decade (2003–2013), and supported subsequently by the results of next-generation DNA/RNA sequencing technologies, was the major unexpected discovery that over 80% of the genome is functionally active [1]. While just 1.5% of the genome encodes proteins, the remaining 98.5% is non-coding. These noncoding regions, once dismissed as “junk” DNA but now understood to be abundantly functional, encompass diverse regulatory units that work both at the genomic and epigenetic levels (for instance, enhancers and *cis* and *trans*, proximal and distal regulatory elements) as well as at the transcriptomic level as transcriptional units that give rise to macro and micro non-coding RNAs. Of the former (macro) ncRNAs, lncRNAs are estimated to be encoded by anywhere from 15,000 to 80,000 distinct *loci* in humans [2–4]. LncRNAs with emerging regulatory functions in different pathways of cellular biology are gaining attention in recent years since they have been functionally associated with a wide range of human diseases, and many of them are now being intensively pursued as potential targets for therapeutics. According to the definition currently adopted by the ncRNA-biology community, lncRNAs are defined as non-protein-coding RNA molecules greater than 500 nucleotides in length [5]. In the two decades that have elapsed since the discovery of their widespread incidence, it has been widely accepted that lncRNAs regulate gene expression at epigenetic, transcriptional, and post-transcriptional levels [6]. Although lncRNAs do not code for any known proteins or long peptides in their classical definition, recent findings have demonstrated that certain lncRNAs translate into micropeptides with regulatory roles in cells [7, 8]. Long non-coding transcripts outnumber protein-coding genes in mammalian genomes (comprising approximately two-thirds of human genes) and, relative to protein-coding genes, are poorly conserved between closely related species and lineages in evolution, more weakly transcribed, and possess striking cell-type and tissue specificity [6]. The occurrence of lncRNAs in numerous disparate biological contexts is commensurate with their profound implications for human health and disease. They are already firmly functionally linked to multiple human diseases, from cancer to autoimmune, inflammatory, and neurological disorders [9]. At the cellular level, dysregulation of lncRNAs affects cell proliferation, cell metabolism, cell differentiation, apoptosis, angiogenesis and metastasis, and genomic instability [10].

**Fig. 1:**
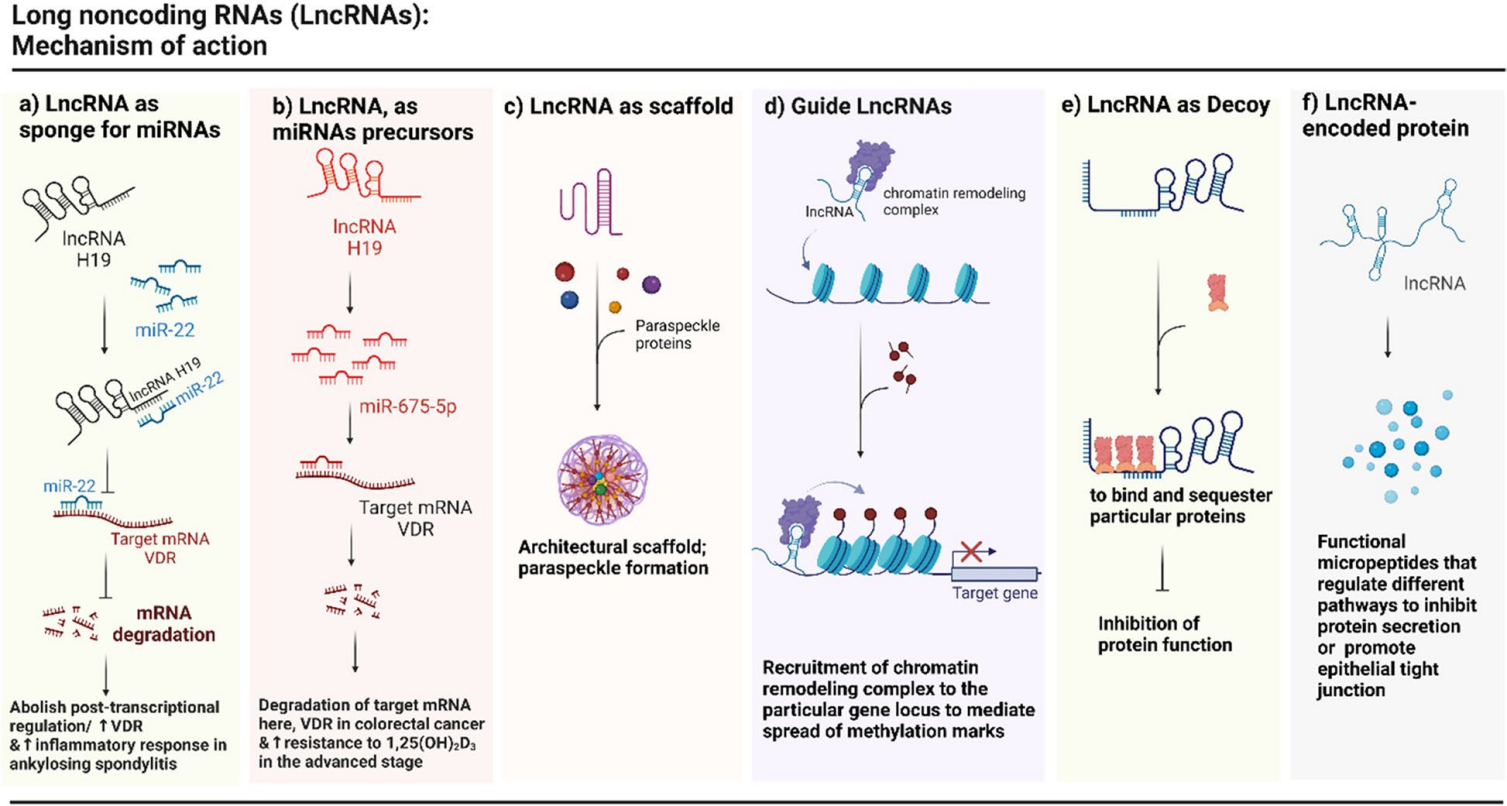
Selected major mechanisms of action of long noncoding RNAs (lncRNAs). **a** LncRNA may function as a sponge or a competing endogenous RNA (ceRNA) [14] for specific miRNAs to consequently nullify the silencing of target mRNAs by those miRNAs. **b** LncRNAs may act as hosts of, and hence be processed into, small RNAs including miRNAs, to promote silencing of the target mRNAs. **c** LncRNAs could assemble a set of proteins to enable a biological event such as suppression or activation of genes or forming nuclear subcompartments (including paraspeckles) by serving as an architectural scaffold. **d** Signal lncRNAs can facilitate the recruitment of proteins into a particular part of the genome for further modification. **e** LncRNA may entrap proteins, such as histone modifying enzymes or transcription factors to block their binding to the specific part of the genome and hence indirectly affect the expression of those proteins’ target genes. **f** Selected lncRNAs may encode micropeptides with regulatory roles in the cells [7, 15]. Created with BioRender.com

LncRNAs are mainly transcribed by RNA polymerase II (Pol II), and similarly to mRNAs, 7-methylguanosine capping and polyadenylation occur at their 5′ and 3′ ends respectively [11], and the majority of lncRNAs in humans are cytoplasmic [12]. Based on their relative position and directionality with respect to nearby and overlapping protein-coding genes, they are categorized into several classes (corresponding to ENCODE’s Gencode biotypes), including but not limited to sense overlapping, antisense, bidirectional promoter sharing, intronic, and intergenic lncRNAs. They act through multiple, highly heterogenous, positive as well as negative, epigenetic as well as post-transcriptional, gene-specific as well as global, mechanisms to regulate gene expression (Fig. 1). Illuminating the molecular mechanisms behind their function provides the opportunity to discover new diagnostic markers and therapeutic targets. Among many other well-characterized roles, lncRNAs can sequester and interact with miRNAs and mRNAs, form RNA–protein complexes, and serve as the host transcripts processed to produce miRNAs and other diverse small RNAs, hence indirectly modulating the expression of target genes. The association of lncRNA, miRNA, and mRNA regulatory networks points to new directions of discovering promising therapeutic targets. The 3D structure of lncRNAs with different domains (protein-binding, other-RNA-binding, DNA-binding, and linkers) can provide a scaffold to assemble multiple proteins into RNA–protein complexes. This can control the sequences of protein recruitment events to modulate gene expression or facilitate the formation of intracellular structures such as nuclear speckles and paraspeckles [13]. Guide lncRNAs direct regulatory factors, such as chromatin modifiers and transcription factors, into specific regions of the genome. LncRNAs, in addition to functioning at the RNA level, have the potential to code for micropeptides, which add an extra layer to regulatory networks [11].

Numerous human conditions and diseases stem from environmental factors or gene-environment interactions. The field of nutrigenomics investigates how food and nutrition interact with individual genes to affect gene expression signatures and hence a person's health and risk of developing diseases [16]. Recent evidence suggests that vitamins and lncRNAs are interconnected through defined regulatory axes. This type of interaction is expected, since lncRNA in eukaryotes, similarly to prokaryotic riboswitches, are thought to be at the forefront of sensing the environment [17, 18]. In this review, we aimed to summarize contemporary peer-reviewed literature that identified specific functional linkages between vitamins and lncRNAs in humans or mammalian models. We first provided a brief overview of the source, metabolism, and function of vitamins in their related sections. We then tabulated and discussed the available research, highlighting any links between vitamins and lncRNAs. To our knowledge, this is the first review that has comprehensively profiled the co-contribution of lncRNAs and multiple vitamins to common pathways, beyond the vitamin D/VDR pathway that has been the focus of prior published studies [19–21].

## Vitamins, a brief history

The necessity of vitamins for human life was widely recognized after it was first comprehensively characterized by Casimir Funk in 1912 [22]. Funk summarized that adding a trace of these “magical substances” to the diet could simply rescue the devastating diseases such as scurvy, rickets, beriberi, and pellagra. He is considered the ‘father of vitamin therapy’ and coined the term “vitamin” (‘vita’ indicative of a vital substance and ‘amine’ as he thought this essential substance is a chemical amine). However, after it was found that the other compounds in this class do not contain necessarily an amine group, it is shortened to ‘vitamin’ regardless of their chemical makeup. Soon afterward, different types, natural sources, and chemical structures of vitamins were identified. Vitamins are generally categorized based on their solubility into fat-soluble (e.g., vitamins A, D, E, and K) and water-soluble vitamins (e.g., B and C) with distinct physical and biochemical characteristics. Their biological roles and association with metabolic pathways and diseases were quickly determined. Vitamins command a vast array of essential biological activities, acting as coenzymes, antioxidants, hormones, and regulating cellular proliferation and coagulation. Following the introduction of molecular biology into the field of nutrition to address different individuals’ responses to nutrition, the branch of molecular nutrition developed. The concept of the lactose operon was proposed by Jacob and Monod (1961) as the first example of gene-nutrition interaction [23] which was then demonstrated by Shapiro et al. (1969) [24]. Now, in the era of high-throughput genomics and multi-omics approaches, a wealth of reports demonstrates how our dietary intake can shape our transcriptome, with potential impacts on health status, taking this topic from its infancy in 20th-century biology of prokaryotes to its blossoming maturity in the post-genomic eukaryotic genetics’ era.

## Functional link between fat-soluble vitamins (A, D, E And K) and long non-coding RNAS

### Vitamin A

Vitamin A is bioavailable in three forms: retinol, retinal, and retinoic acid. It has long been explored as a key illustration of the regulation of gene expression by a nutrient. Retinol has a number of downstream metabolites, namely all-*trans* retinoic acid (AtRA), and 9- or 11-*cis* retinoic acids. These metabolites can activate their cognate receptors in the nucleus, thus regulating expression of target genes [25]. This nutrient and its metabolites exert pleiotropic impacts in a variety of tissues affecting developmental processes, proliferation and apoptosis of cells and metabolic pathways [25]. The interactions between vitamin A (and/or its metabolites) and lncRNAs have been studied in different pathological conditions, such as autism, multiple types of brain disorders, congenital scoliosis, and several cancers (Table 1).

**Table 1:** Evidence of specific functional relationships between vitamin A (including its key derivatives and related compounds) and long noncoding RNAs (lncRNAs)

**Vitamin**	**LncRNA(s) studied in the project**	**System: Patients / cell line(s) / animal model**	**Highlighted lncRNA(s)/pathway(s)/target(s)/ partners/ interactions**	**Disease context/ implication for human disease**	**Main result(s)**	**Ref**
**Vitamin A metabolism-associated disorders in pregnancy and early development**						
**A**	NONRATT021475.2	Pregnant rats exposed to valproic acid to induce ASD model in offspring. Expression analysis of the lncRNA and mRNA in the hippocampus	NONRATT021475.2/ Desert hedgehog (Dhh)	Autism-Spectrum Disorder	Improvement of valproic acid-induced autism-like behaviors through NONRATT021475.2/Dhh axis	[26]
**Vitamin A and Retinoic Acid (RA)**	SULT1C2A	Rat model of vitamin A deficiency (VAD)‐induced Congenital Scoliosis (CS) Human HEK‐293T and H9C2 cells	lncRNA SULT1C2A‐rno‐miR‐466c‐5p‐Foxo4 axis	Congenital Scoliosis	Vitamin A deficiency during pregnancy could induce CS in offspring. Key finding: VAD dysregulated the lncRNA SULT1C2A‐rno‐miR‐466c‐5p‐Foxo4 axis during somitogenesis	[27]
**A**	749 mRNAs, 56 miRNAs, 685 lncRNAs, and 70 circRNAs were differentially expressed	Rat embryos	pathways enriched in VAD-CS pathogenesis: Wnt, PI3K-ATK, FoxO, EGFR, and mTOR	Congenital Scoliosis	In vitamin A deficiency-induced CS, specific lncRNA networks are dysregulated	[28]
**Vitamin A metabolism-associated disorders during adulthood**						
**A**	high-FCR (H) vs. low-FCR (L): 300 differentially expressed (DE) genes. 40/300 were lncRNAs. 25/40 lncRNAs were significantly correlated with 125 DE protein-coding genes	Yorkshire pig [*n* = 236, for feed conversion ratio (FCR) assessment] Liver; RN-seq	_	Pig breeding [Feed efficiency (FE) in pig]	DE genes, including lncRNAs, were enriched in vitamin A, fatty acid, and steroid hormone metabolism pathways	[29]
**Vitamins A (AtRA) and D**	LINC00595, SBF2-AS1 (A.fumigatus) and RP11-588G21.2, RP11-394l13.1 (C.albicans detectable in the early phase of infection; CTD3128G10.6 and SRP as potential markers for *E. coli* infection	Human monocytes Pathogenic fungi: *C. albicans* (SC5314) & *A. fumigatus* (AF293) Bacteria: *E. coli* (isolate 018:K1:H7)	_	Fungal & bacterial infection	To investigate the roles of vitamins A and D during infection, specific ncRNAs were implicated in infection response MarkersLINC00595, SBF2-AS1 (*A.fumigatus*), and RP11-588G21.2, RP11-394l13.1 (*C.albicans*) were detectable in the early phase of infection, hence may be potential therapeutic targets	[30]
**AtRA**	Long intergenic noncoding RNA-rat brain expressed (LINC-RBE)	Primary hippocampal neuronal cell culture from adult (16 weeks) male rat (Rattus norvegicus)	_	Neuronal function	AtRA is involved in transcriptional upregulation of lncRNA expression (here LINC-RBE)	[31]
**AtRA**	hTR	human ovarian cancer cell lines	_	Ovarian carcinoma cells	In AtRA-treated ovarian carcinoma cells, expression of the telomerase components, hTERT and hTR was reduced, implying that ATRA may act by suppressing telomerase activity to inhibit cell growth	[32]

#### Vitamin A metabolism-associated disorders in pregnancy and early development

Vitamin A supplementation may help to manage and treat autism-like behaviors induced by prenatal exposure to the anticonvulsant drug valproic acid (VPA) in a rodent model, by acting through the lncRNA/mRNA axis NONRATT021475.2/Dhh in pregnant rats [26]. Vitamin A plays a key role in central and peripheral nervous system embryonic development, and several studies implicated vitamin A supplementation as a treatment for autism spectrum disorder (ASD) [33, 34]. Prenatal exposure to VPA decreased serum levels of vitamin A, and significantly altered the expression of more than 200 lncRNAs and 300 mRNAs. RT-PCR confirmed the upregulation of 4 lncRNAs and 6 mRNAs participating in neural function and developmental processes, through lncRNA-mRNA co-expression networks, such as NONRATT021475.2-Desert hedgehog (Dhh). Besides, vitamin A supplementation was able to restore that regulatory network, reducing the autism-like behaviors induced by VPA in the hippocampus of offspring [26].

Congenital scoliosis (CS) is a sideways curvature of the spine caused by abnormal vertebrae growth during embryogenesis [35]. Vitamin A is known to play important roles in the pathogenesis of CS; maternal vitamin A deficiency induces CS deformities in rat offspring [36]. The retinol-retinoic acid metabolism pathway is impaired in a rat model of congenital kyphoscoliosis [37]. A vitamin A deficiency-induced congenital scoliosis rat model showed a dynamic correlation between lncRNA SULT1C2A, rno‐miR‐466c‐5p, and Foxo4 expression, where SULT1C2A regulates Foxo4 by targeting rno-miR-466c-5p through PI3K-ATK signaling [27]. Specifically, rno-miR-466c-5p downregulates Foxo4, reducing AKT and p85 (the regulatory subunit of PI3K) phosphorylation. These effects are reversed by SULT1C2A (whose expression is increased in CS) which, by acting as a ceRNA of rno-miR-466c-5p, upregulates Foxo4 [27]. Similarly, there is coordinated lncRNA/mRNA network deregulation in vitamin A deficiency-induced congenital scoliosis. The association of mRNAs and ncRNAs in the pathogenesis of CS has been demonstrated by transcriptome sequencing [28].

#### Vitamin A metabolism-associated disorders during adulthood

Regulation of lncRNAs by vitamins, as an integral part of vitamin-regulated gene expression programs, is also supported by the recent demonstration of 300 differentially expressed (DE) transcripts, including 232 protein-coding gene mRNAs, 28 endogenous *cis*-antisense transcripts, and 40 lncRNAs, correlated with vitamin A, fatty acid, and steroid hormone metabolism. That study also revealed that vitamin A metabolism in liver affects feed efficiency in pigs [29]. RNA-seq of monocytes demonstrated that vitamins A and D modulate transcriptional regulation of host ncRNAs in fungal and bacterial infections, regulating the pro-inflammatory response. The lncRNAs LINC00595, SBF2-AS1, RP11-588G21.2, and RP11-394l13.1 were identified in that study as potential biomarkers of and putative therapeutic targets in fungal infection [30]. The vitamin A metabolite all-trans retinoic acid (AtRA) transcriptionally upregulates the intergenic lncRNA, LINC-RBE, in adult rat hippocampal neurons [31]. Studies pointing to a functional relationship between vitamin A and lncRNAs are summarized in Table 1.

### Vitamin D and The Vitamin D Receptor (VDR)

**Fig. 2:**
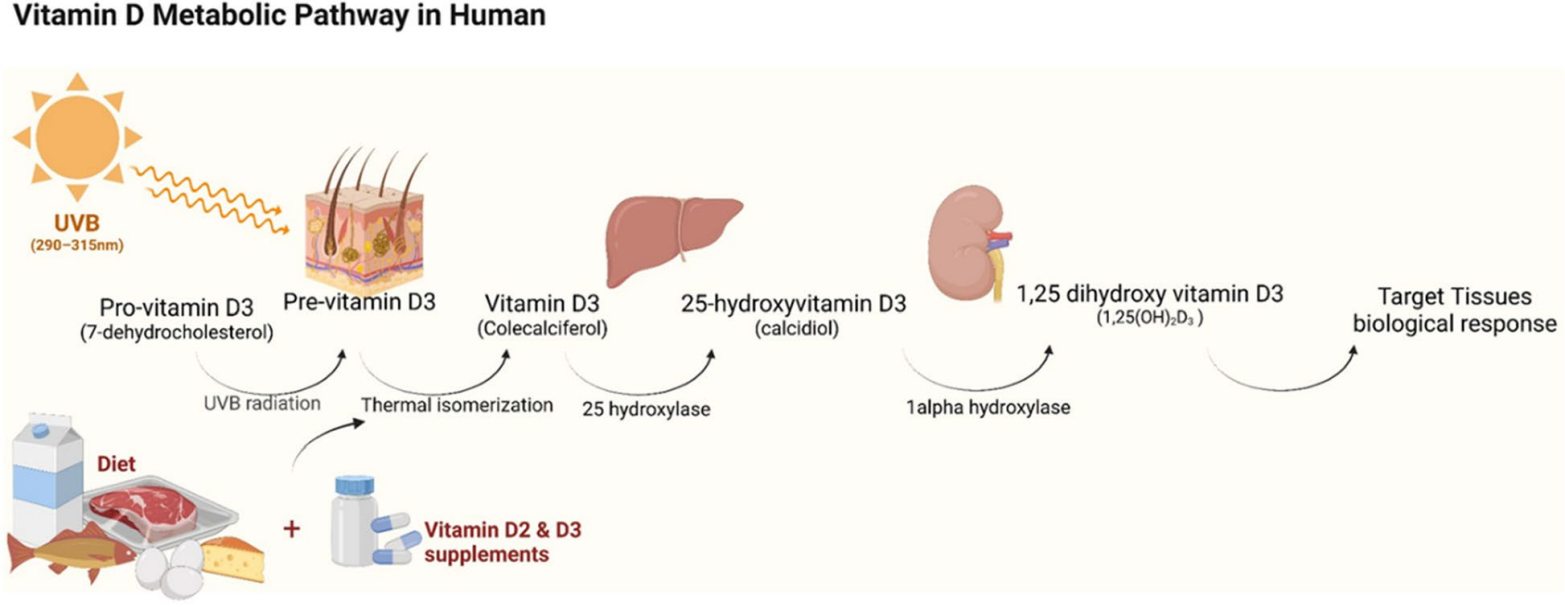
Vitamin D is obtained through two main sources: cutaneous synthesis and oral intake. UVB radiation (290–315 nm) photolyzes 7-dehydrocholesterol to pre-vitamin D3 which in turn is converted to vitamin D3 by isomerization through a thermo-sensitive reaction in the epidermis. Upon binding to vitamin D binding protein (DBP), the synthesized vitamin D3 traverses the systemic circulation to the liver. In the liver, vitamin D3 is hydroxylated to produce 25-hydroxyvitamin D3 [25(OH)D3], the major circulating vitamin D metabolite. DBP transports 25(OH)D3 to the kidney where it is converted to calcitriol (1,25-dihydroxyvitamin D3 [1,25(OH)_2_D_3_]), a potent steroid hormone and the active metabolite of the vitamin D. Created with BioRender.com

**Fig. 3:**
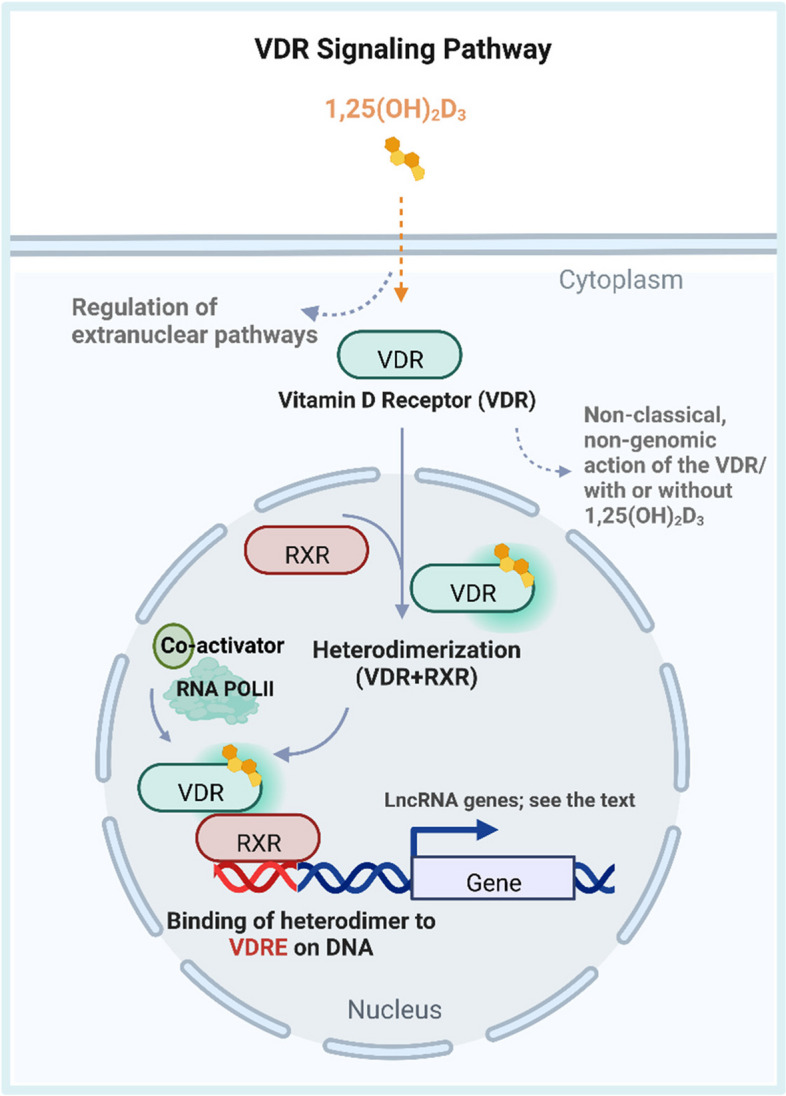
Tracing the fate of 1,25(OH)_2_D_3,_ the active form of vitamin D in target cells. The lipophilic nature of 1,25(OH)_2_D_3_ helps it to passively cross cell membrane without a transporter and bind to VDR in the cytoplasm and/or the nucleus. As a nuclear transcription factor, VDR regulates the transcription of target genes in a ligand-dependent manner. Upon binding to 1,25(OH)_2_D_3_, VDR enters the nuclei and heterodimerizes with the retinoid X receptor (RXR). The active VDR/RXR complex is then able to bind specific consensus sequences, vitamin D response elements (VDREs), at the promoters of its direct target genes. It also initiates the recruitment of co-activators or co-repressors to modulate the target genes so as to maintain homeostasis. Beyond VDR, its classical partner, 1,25(OH)_2_D_3_ can also bind to membrane-embedded receptors and affect cytoplasmic signaling cascades through its non-transcriptional activities [39]. Direct or indirect downstream lncRNA targets of the vitamin D/VDR signaling pathways include AS-HSD17β2 in prostate cancer [40], H19 in colon cancer [41], lncBCAS1-4_1 [42] and TOPORS-AS1 [43] in ovarian cancer, LUCAT1 in oral squamous cell carcinoma (OSCC) [44], MEG3 in colorectal cancer (CRC) [45], MALAT1 in coronary artery disease (CAD) [46], and HOTAIR in multiple sclerosis [47]. Created with BioRender.com

Vitamin D exerts its role through modulating the activity of the vitamin D receptor (VDR). This receptor is a nuclear-receptor transcription factor, one of over 40 human transcription factors that, upon binding the cognate receptor, translocate from the cell surface through the cytoplasm into the nucleus, where they bind the promoters of the direct-target genes that they activate and/or repress. Establishment of the complex between VDR and its ligand, the active form of vitamin D, i.e. vitamin D 1,25(OH)_2_D_3_, leads to the translocation of the activated VDR from the cytoplasm into the nucleus and hence to the direct regulation of expression of hundreds of genes, including those involved in infection response and immune disorders [38], through the binding of VDR to the promoters and regulatory elements of those genes. Figure 2 represents an overview of vitamin D metabolic pathways. Figure 3 shows the molecular pathways through which VDR affects gene expression in target cells.

**Fig. 4:**
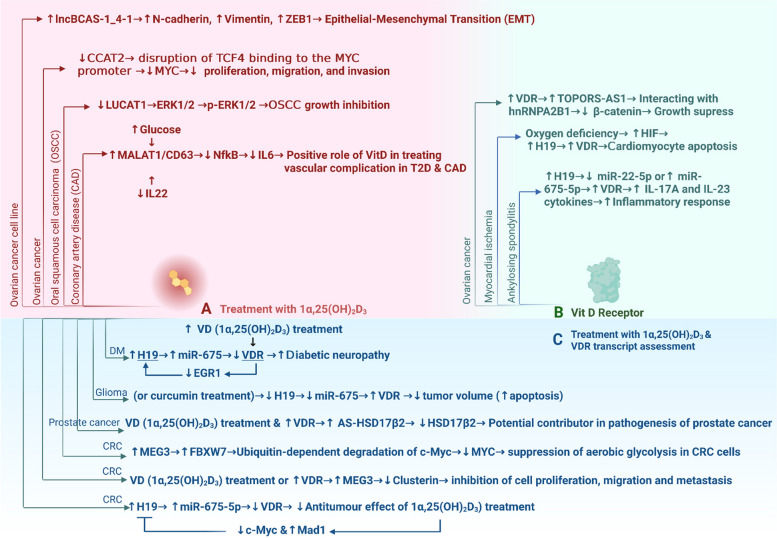
Molecular pathways and axes in human disease, inferred from the published literature coupling the vitamin D / VDR pathway to long noncoding RNAs (lncRNAs). Since 1,25(OH)_2_D_3_ can affect intracellular pathways via different VDR or/and non-VDR partners, we categorize the pathways into those that are suggested after treatment with 1,25(OH)_2_D_3_ (A, the top left), those that focused on VDR itself (B, induction or gene expression assessments) (top right), and those that jointly evaluated the effect of 1,25(OH)_2_D_3_ treatment and VDR at the same time (C, bottom). See Table 3 for details. CRC: Colorectal cancer; DM: Diabetes Mellitus. Created with BioRender.com

The interactions between vitamin D/VDR and lncRNAs have been assessed in different disease contexts, including cancer, neuropsychiatric disorders and coronary artery disease. Evidence of the specific functional relationships between vitamin D/VDR and lncRNAs is summarized in Table 2. Notably, the protective role of vitamin D against breast cancer through lncRNA-mediated pathways has been elucidated in detail [19, 20]. Here we provided an update of current research and extracted the main regulatory axes with vitamin D/VDR and lncRNAs as reported there (Table 2). Moreover, we consider the fact that although vitamin D and VDR are recognized as faithful classical partners, either one can modulate intracellular activities via other pathways, independently of each other. Hence, we separated studies into those that investigated the effect of 1,25(OH)2D3 treatment only, those that focused on VDR itself (by induction or gene expression assessments), and those that jointly evaluated the effect of 1,25(OH)2D3 treatment and VDR at the same time. Molecular pathways and axes through which vitamin D and VDR are linked to the lncRNAs are shown in Fig. 4. MALAT1, the snoRNA host genes SNHG16 and SNHG6, LINC00346 and LINC00511 are among VDR-associated lncRNAs identified through an in silico approach in breast cancer [48]. Further experiments have shown upregulation of VDR, MALAT1 and LINC00511 in breast tumors relative to nearby non-cancerous samples, and associations between clinicopathological data and expression of VDR-associated lncRNAs [48]. These lncRNAs have also been examined in Parkinson's disease [49], bipolar disorder [50], schizophrenia [51], and epilepsy [52].

**Table 2:** Evidence of specific functional relationships between the vitamin D / Vitamin D Receptor (VDR) pathway and long noncoding RNAs (lncRNAs)

**Compound**	**LncRNA(s) studied in the project**	**System: Patients / cell line(s) / animal model**	**Highlighted lncRNA(s)/ pathway(s)/ target(s)/ partners/ interactions**	**Disease context/ implication for human disease**	**Main result(s)**	**Ref**
**Cancer**						
**Vitamin D**	AS-HSD17β2 (RP11 510J16.5)	Human keratinocyte cell line: (HaCaT) Human prostate cancer cell lines: PC3, LNCaP, CWR22	HSD17β2/ AS-HSD17β2 (Hydroxysteroid 17-Beta Dehydrogenase 2)	Prostate cancer	AS-HSD17β2 is a potential directly regulated target of vitamin D. It attenuates HSD17β2 expression, HSD17β2is involved in the VDR pathway and steroid metabolism	[40]
	LUCAT1	human oral squamous cell carcinoma (OSCC) cell lines: CAL27 and SCC9 OSCC patient tumor samples and adjacent noncancerous (ANC) samples	LUCAT1-MAPK signaling pathway	Oral squamous cell carcinoma (OSCC)	Through the MAPK pathway, vitamin D suppresses the growth of oral squamous cell carcinoma by inhibiting the lncRNA LUCAT1 This study helps to mechanistically explain how vitamin D may suppress the progression of oral cancer	[44]
	lncBCAS1-4_1	Human ovarian cancer cell line SKOV3	Link between EMT and vitamin D signaling via the lncBCAS1-4_1-lncRNA	Ovarian cancer	In a study of 1α,25(OH)_2_D_3_ impact on expression of lncRNAs, lncBCAS1-4_1 suggested a link of vitamin D signaling and EMT	[42]
	CCAT2	The epithelial ovarian carcinoma (EOC) cell lines SKOV3 and A2780	VitD/CCAT2 axis	Ovarian Cancer	Vitamin D inhibits ovarian cancer cell growth via downregulation of lncRNA CCAT2	[53]
	MEG3	371 colorectal cancer patients who underwent curative resection RKO, SW1116, HT29, HCT116, LoVo, SW620, SW480 and 293T	VDR/MEG3/Clusterin	Colorectal cancer (CRC)	• Down-regulation of MEG3 was observed in CRC tissues and associated with poor prognosis • MEG3 may act through the VDR/MEG3/Clusterin pathway MEG3 inhibited CRC metastasis and proliferation	[45]
	lnc-CYP24A1-3:1, and lnc-TSHZ2-19:1 (Novel lncRNAs related to CYP24A1 and PFDN4 genes in colorectal cancer (CRC))	Colon cancer cell lines: HCT-116 and HT-29	CYP24A1	Colorectal Cancer (CRC)	CYP24A1, PFDN4, lnc-CYP24A1-3:1, and lnc-TSHZ2-19:1 are putative novel diagnostic markers for CRC. Vitamin D regulates expression of these genes	[54]
	MEG3	80 CRC samples and corresponding adjacent normal mucosal samples human CRC cell lines: DLD-1 and RKO	Vitamin D/MEG3/ c-myc	Colorectal cancer (CRC)	Vitamin D-activated long non-coding RNA MEG3 suppresses glycolysis in CRC by enhancing c-Myc degradation	[55]
	H19	Human cell lines: SH‐SY5Y and SNB‐19 cells Rat brain tissues	H19/miR-675/vitamin D receptor (VDR)	Glioma	• A negative feedback loop of H19/miR-675/VDR has been shown in the development of glioma • The effect of [curcumin and also] 1,25(OH2 D3) was assessed	[56]
	H19	PBMCs of newly diagnosed ALL patients (For gene expression analysis) & Serum (for 25-hydroxy vitamin D measurement)	_	Acute Lymphoblastic Leukemia (ALL)	Gene expression analysis showed concurrent downregulation of VDR and H19 expression in ALL patients vs control group (potential implication for H19 as both a biomarker and a functional contributor)	[57]
**VDR**	H19	Patients with colon cancer Cell lines: HT-29 and DLD-1	VDR/H19/miR-675-5p	Colon cancer	Overexpression of H19 increases resistance to 1,25(OH)_2_D_3_ by downregulation of VDR through miR-675-5p. VDR signaling may suppress H19 via the C-Myc/Mad-1 network	[41]
	TOPORS-AS1	Fresh tumor samples of 266 patients with ovarian cancer Six ovarian cancer cell lines: (IGROV1, SKOV3, OVCAR3, OVCAR4, OVCAR5, OVCAR8)	Wnt/β-catenin pathway	Ovarian cancer	The lncRNA TOPORS-AS1 was highlighted as a potential tumor suppressor by interruption of the Wnt/β-catenin signaling. VDR could positively regulate TOPORS-AS1	[43]
	LINC00346; LINC00511; MALAT1; SNHG16; SNHG6	75 breast tumor samples (invasive ductal carcinoma of breast) and their adjacent noncancerous tissues (ANCTs)	_	Breast cancer	• VDR, MALAT1, and LINC00511 were upregulated in tumors vs. ANCT • SNHG16 and LINC00511 expression levels correlated with nuclear grade. LINC00346 level correlated with tubule formation. SNHG16 and SNHG6 expression levels correlated with family history of cancer. VDR expression correlated with progesterone receptor status • *FOKI* polymorphism was associated with over-expression of VDR • *FOKI* variants were associated with expression levels of MALAT1 and SNHG16 in non-cancerous tissues • CDX2 variants were associated with expression levels of SNHG16 in ANCTs. SNHG16 expression significantly correlated with vitamin D levels	[48]
	MALAT1	Computational identification of lncRNAs that modulate VDR signaling in BC	_	Breast cancer	• MALAT1 was among lncRNAs inferred to affect VDR signaling in BC	[58]
	MALAT1, SNHG16, SNHG6, LINC00346, LINC00511	32 pairs of lung cancer tissues and adjacent non-cancerous tissues (ANCTs)	LINC00346 SNHG6	Lung cancer	• VDR and LINC00346 were downregulated in male tumor tissues vs matched ANCT	[59]
**Neurological and brain disorders**						
**Vitamin D**	HOTAIR	42 patients with relapsing–remitting MS Cell line: THP-1 cells	_	Multiple Sclerosis	The lncRNA HOTAIR was overexpressed in the PBMCs of MS patients with VD-deficiency compared with healthy controls	[47]
	LncRNAs previously known to be relevant to VDR (LINC00511, LINC00346, SNHG6 and SNHG16)	Peripheral blood of 40 epileptic patients and 39 healthy subjects	_	Epilepsy	• Expression of VDR-related lncRNAs (including LINC00511, LINC00346, SNHG6, and SNHG16) was assessed in epileptic patients • SNHG16 was upregulated in male patients vs. male controls • LINC00511 was upregulated in female patients vs. female control • Significant association of SNHG6 and SNHG16 expression with gender • Significantly co-expressed correlated gene pairs were found: [SNHG6 and SNHG16], [SNHG6 and LINC00346], [SNHG16 and LINC00346], [SNHG16 and LINC00511] • Inverse correlation between expression of LINC00346 and vitamin D levels only in male epileptic patients	[52]
**VDR**	SNHG6, SNHG16, LINC00346	Parkinson's disease patients	_	Parkinson's disease (PD)	• Expression of VDR and SNHG6, SNHG16, and LINC00346 was assessed in PD • SNHG6 and VDR differentiate PD patients from controls	[49]
	VDR-associated lncRNAs (SNHG6, CYP27B1, MALAT1, Linc00346, LINC00511)	Peripheral blood of BD patients vs. healthy individuals	_	Bipolar disorder (BD)	• Upregulation of SNHG6, CYP27B1, MALAT1, Linc00346, and VDR in total BP patients vs. control groups • Upregulation of SNHG6, CYP27B1, MALAT1, Linc00346, VDR in BP males vs. normal male controls • Upregulation of SNHG6 in BP females. vs. normal female controls • These VDR-associated lncRNAs are possible biomarkers for BD and should be investigated for causality in its etiology	[50]
	SNHG6, LINC00346, LINC00511	Peripheral blood of schizophrenia patients vs. healthy individuals	_	Schizophrenia (SCZ)	• Upregulation of SNHG6 and LINC00346in SCZ patients vs. controls • These VDR-associated lncRNAs are possible biomarkers for SZ and should be investigated for causality in its etiology	[51]
	SNHG6 and LINC00511	Circulating blood transcriptome of ASD patients compared with normal controls	_	Autism Spectrum Disorder (ASD)	Gene expression assessment of VDR, CYP27B1, SNHG6, and LINC00511 showed upregulation of CYP27B1 & downregulation of in ASD patients vs controls, implying a role in ASD	[60]
**Cardiovascular diseases**						
**Vitamin D**	MALAT1	PBMCs of coronary artery disease (CAD) patients & non-CAD (NCAD) participants	Expression correlation between MALAT1/CD36/IL-22	Coronary Artery Disease (CAD)	A significant correlation was found, implying a protective role of vitamin D against vascular complications in CAD	[46]
**VDR**	H19	Infarcted heart tissue of H19 KO mice	H19-VDR axis	Myocardial ischemia	Investigation of the role of H19 in myocardial infarction in vivo and under oxygen deficiency in vitro showed that H19 regulates cardiomyocyte apoptosis and cardiac inflammation through the VDR pathway	[61]
**Diabetes**						
**Vitamin D**	_	Endothelial Progenitor Cells (EPCs) and control cells from bone marrow (BM)	_	Endothelial cell function in type 2 diabetes mellitus (T2D)	1,25(OH)_2_D_3_ enhances endothelial progenitor cell (EPC) function through a competing endogenous RNA (ceRNA) network	[62]
	LINC01173	200 newly diagnosed untreated T2DM and 200 healthy controls	_	Type 2 diabetes mellitus (T2DM)	Overexpression of lncRNA LINC01173 in the blood of vitamin-D deficient T2DM cases was significant compared to insufficient and sufficient T2DM groups	[63]
**VDR**	H19	Human subjects: Renal tissues of 54 Diabetes Mellitus patients [29 DM patients with diabetic nephropathy (DN) and 25 without DN] Cell lines: CIHP-1/HEK 293 cells	H19/miR-675/EGR1	Diabetic Nephropathy (DN)	An inhibitory loop including H19, miR‐675, VDR, and EGR1 was demonstrated in the pathological mechanism of DN	[64]
**Other pathologies**						
**VDR**	H19	Peripheral Blood Mononuclear Cells (PBMCs) of AS [5 AS patients and 5 healthy controls (HCs)]	H19-miR22-5p/miR675-5p-VDR-IL-17A/IL-23 signaling	Ankylosing Spondylitis (AS)	H19-miR22-5p/miR675-5p-VDR-IL-17A/IL-23 signaling pathways are implicated in the pathogenesis of AS	[65]
	Differential expression analysis of miRNAs, lncRNAs, and mRNAs (Microarray analysis)	Sprague Dawley rats	miR-98/VDR 5 lncRNAs (NR_046246.1, NR_046239.1, XR_086062.1, XR_145872.1 and XR_146737.1), may play important roles in regulating the CN-induced osteogenic differentiation of MSCs	Mesenchymal Stem Cells (MSCs) osteogenic differentiation	To interrogate the role of ( +)-cholesten-3-one (CN) in osteoblastic differentiation of (MSCs), DE analysis was performed and showed enrichment in the VDR pathway. Key finding: miR-298 could enhance the osteogenic differentiation of MSCs via the VDR pathway	[66]
	SNHG6, SNHG16	Peripheral blood	_	COVID -19	Expression assessment of VDR, VDR-associated lncRNAs (including SNHG16, SNHG6, LINC00346, and LINC00511), and CYP27B1 in COVID-19 patients versus healthy subjects showed plausible joint roles of SNHG16 and VDR inCOVID-19 infection	[67]

Figure 4 shows pathways through which vitamin D and VDR are linked to specific lncRNAs.

In addition to the several reports of dysregulation of VDR-associated lncRNAs in different disorders, other studies have revealed functional evidence of a regulatory relationship between vitamin D/VDR and specific lncRNAs (Table 3). For instance, Kanemoto et al. combined in silico and expression assays in human cell lines to identify vitamin D-regulated non-coding RNAs. Their approach led to the identification of four lncRNAs which are directly regulated by this vitamin. These findings have been confirmed by identification of consensus VDR-binding motifs in the coding regions of these lncRNAs. Notably, the antisense transcript from the HSD17β2 *locus* (AS-HSD17β2) is among these directly vitamin-D-regulated lncRNAs. This transcript has been shown to attenuate HSD17β2 expression [40]. lncBCAS1-4_1 is an lncRNA functionally associated with vitamin D signaling in ovarian cancer cells, and is involved in the epithelial-mesenchymal transition in ovarian cancer [42]. Moreover, expression of MALAT1 is affected by vitamin D status in patients with coronary artery disease and healthy subjects [46]. In type 2 diabetes mellitus (T2DM), the lncRNA LINC01173 was upregulated in the blood of vitamin-D deficient T2DM cases, relative to controls [63]. Additional examples of this type of correlative finding are presented in Table 3.

**Table 3:** Functional evidence of vitamin D and vitamin D Receptor (VDR) signaling pathways linked to long noncoding RNAs (lncRNAs)

	**Diseases/Cell lines**	**LncRNA**	**Vitamin D & VDR pathway**	**Comments**	**Ref**
**Treatment with 1α,25(OH)**_**2**_**D**_**3**_	Ovarian cancer cell line	LncBCAS1-4_1	↑lncBCAS-1_4-1 → ↑N-cadherin, ↑Vimentin, ↑ZEB1 → Epithelial-Mesenchymal Transition (EMT)	Induced expression of lncBCAS-1_4-1 disrupts the inhibitory effect of 1a,25(OH)2D3 in SKOV3 cell line (Ovarian Cancer)	[42]
	Ovarian cancer	CCAT2	VD (1α,25(OH)2D3) treatment → ↓CCAT2 → dysruption binding TCF4 to MYC promoter → ↓ MYC → ↓ proliferation, migration and proliferation	By targeting CCAT2, vitamin D suppressed proliferation in ovarian cancer	[53]
	Oral squamous cell carcinoma	LUCAT1	VD (1α,25(OH)2D3) treatment → ↓LUCAT1 → ERK1/2 → p- ERK1/2 → OSCC growth inhibition	VD treatment of human oral squamous cell carcinoma cell lines led to inhibition of cell proliferation by decreasing the expression of LUCAT1 which subsequently inhibited MAPK pathway activation	[44]
	Coronary artery disease	MALAT1		VD supplementation increased the level of MALAT1 and decreased the level of IL22 and IL6 in coronary artery disease (CAD) patients	[46]
**Vitamin D Receptor**	Ovarian cancer	TOPORS-AS1	↑VDR → ↑TOPORS-AS1 → interacting with hnRNP A2B1 → ↓β-catenin	Increased expression of TOPORS-AS1 induced β-catenin degradation and suppressed the Wnt/β-catenin signalling pathway. This inhibitory effect is dependent on hnRNP A2B1.over-expression of VDR positively affects the expression of TOPORS-AS1	[43]
	Myocardial ischemia	H19	Oxygen deficiency → ↑HIF → ↑H19 → ↑VDR → Cardiomyocyte apoptosis	lncRNA H19 has a key role in post-myocardial ischemia Following overexpression of HIF in hypoxic conditions, the expression of H19 and VDR increases in endothelial cells, cardiac fibroblasts, and cardiomyocytes	[61]
	Ankylosing spondylitis		↑H19 → ↓ miR-22-5p or ↑ miR‐675-5p → ↑VDR → ↑ IL-17A and ↑IL-23 cytokines → ↑Inflammatory response	H19 may mediate the inflammatory process in ankylosing spondylitis (AS):as a ceRNA to compete with VDR mRNA for miR-22, or act through its derivative, miR‐675 modulating downstream Wnt/β-catenin signalling	[65]
**Treatment with 1α,25(OH)**_**2**_**D**_**3**_** & VDR transcript assessment**	Diabetes Mellitus	H19		Through a negative feedback loop, H19 downregulates the expression of VDR via upregulation of miR‐675. EGR1 expression diminishes subsequently and inhibits H19 expression in diabetic nephropathy (DN) among patients with diabetes mellitus (DM). Treatment with 1,25D3 and EGR1 downregulated VDR at mRNA and protein levels	[64]
	Glioma		Curcumin or VD (1α,25(OH)2D3) treatment → ↓ H19 → ↓ miR‐675 → ↑ VDR → ↓tumour volume (↑ apoptosis)	Curcumin and 1,25‐dihydroxyvitamin D VD (1α,25(OH)2D3) suppress H19 and miR‐675, leading to creased VDR expression with subsequent tumor volume shrinkage. VDR negatively regulates H19	[56]
	Prostate cancer	AS-HSD17β2	VD (1α,25(OH)2D3) treatment & ↑VDR → ↑AS-HSD17β2 → ↓HSD17β2	AS-HSD17β2 is a direct target of VD (1α,25(OH)2D3). VD-bound VDR induced AS-HSD17β2 in HaCaT (human keratinocyte cell line) and two prostate cancer cell lines (CWR22 and PC3 cells)	[40]
	Colorectal cancer	MEG3	VD (1α,25(OH)2D3) treatment or ↑VDR → ↑MEG3 → ↑ FBXW7 → ubiquitin-dependent degradation of c-MYC protein → ↓ c-MYC target genes involved in glycolysis pathway such as lactate dehydrogenases A, pyruvate kinase muscle 2 and hexokinase 2 (suppression of aerobic glycolysis in CRC cells)	Vitamin D (1,25(OH)2D3) treatment or overexpression of VDR upregulates MEG3 in human CRC cell lines. It then upregulates the FBXW7, encoding one of the subunits of ubiquitin protein ligase. This may lead to the degradation of c-MYC protein and consequent loss of expression of c-MYC target genes	[55]
			VD (1α,25(OH)2D3) treatment or ↑VDR → ↑MEG3 → ↓Clusterin → inhibition of cell proliferation, migration and metastasis	MEG3 overexpression in CRC cells causes downregulation of clusterin with subsequent inhibition of cell proliferation, migration and metastasis. 1α,25-(OH)2D and vitamin D receptor (VDR) could induce expression of MEG3	[45]
		H19		Through the C-Myc/Mad-1 axis, VDR signaling downregulated the expression of H19. Upregulation of H19 induce resistance to the antitumor effect of 1,25(OH)2D3 in CRC cells	[41]

### Vitamins E and K

Both vitamin E and vitamin K are fat-soluble vitamins that are absorbed by the small intestine via lipid transporters and micelles. In the blood, they are carried by lipoproteins and delivered to a variety of tissues. The liver plays a key role in recognizing and metabolizing these vitamins. The excess or non-essential forms of these vitamins are excreted by the body through a series of oxidation reactions. Bioavailability and nutritional requirements of these vitamins vary depending on age, gender, genetics, lifestyle, and smoking [68–70].

In its natural form, vitamin E consists of four tocopherols (αT, βT, γT, δT) and four tocotrienols (αTE, βTE, γTE, δTE) with α-tocopherol being the dominant isoform that our body retains [69]. In the cells, vitamin E metabolites, along with vitamin C, neutralize free radicals, nitrogen oxides, and other electrophilic mutagens. Besides their antioxidant activities, non-antioxidant gene regulatory actions have been demonstrated for them [71]. The activity of protein kinase C is modulated by α-tocopherol, hence broadening the latter’s role beyond antioxidant functions [72]. Modulation of key signaling pathways such as MAPK, PI3K/Akt/mTOR, Jak/STAT, and NF-κB, with anti-inflammatory, immunoregulatory, neuroprotective, anti-proliferative, pro-apoptotic, and anti-angiogenetic outcomes following vitamin E intake, is now documented [73]. Through gene expression profiling methods such as microarray and RNA sequencing, in vivo and in vitro vitamin E intervention studies demonstrated that it affects the expression profile of diverse mRNAs and microRNAs in the liver and potentially in extrahepatic tissues.

To the best of our knowledge, no published data supports the contention that vitamin E may also function through a lncRNA-dependent pathway. However, alpha-tocopherol can prevent ferroptosis, a type of iron-dependent programmed cell death associated with blood and neurological diseases, ischemia–reperfusion injury, kidney injury, inflammation, and cancer [74]. Ferroptosis-related lncRNAs have been reported in a wide range of cancers, including hepatocellular carcinoma [75], head and neck squamous cell carcinoma [76], colorectal cancer [77], and stomach adenocarcinoma [78]. Future studies are expected to define vitamin E/ferroptosis-related lncRNAs axes [79]. Vitamin E is a known modulator of specific miRNA expression [80], and due to the prevalence of integrated miRNA-lncRNA-mRNA regulatory networks, future studies will almost certainly define vitamin-E-dependent examples of these additional regulatory axes.

The Vitamin K group of hydrophilic naphthoquinone compounds mainly features two forms: K1 (phylloquinone) and K2 (menaquinones) [81]. It is an essential cofactor for the post-translational modification of particular proteins involved in bone metabolism and blood coagulation like prothrombin and factors VII, IX, and X. Vitamin K regulation of gene expression has been abundantly documented in the past two decades. Binding of VitK2 to the steroid and xenobiotic receptor (SXR) affects the expression of key genes involved in bone homeostasis, including alkaline phosphatase, osteoprotegerin, and genes involved in in extracellular matrix formation [82]. Vitamin K, through the pregnane x receptor (PXR), alters the expression of drug metabolism-related genes MDR1 and CYP3A4 in the intestine [83]. Emerging evidence suggests that vitamin K may also act through a lncRNA-dependent pathway. Growth arrest-specific gene 6 (GAS6), a vitamin K-dependent protein, is negatively correlated with its putative *cis*-antisense regulator, the lncRNA GAS6-AS, in breast cancer [84]. There is an increase in GAS6 expression following intravenous injection of vitamin K1 in non-warfarin treated patients[85]. Hence, vitamin K may affect the expression of the lncRNA GAS6-AS, a regulator of GAS6. Furthermore, coagulation factor X (FX), another vitamin K-dependent protein, may help recruit tumor-associated macrophages in glioblastoma multiforme FX is regulated by the lncRNA CASC2c and synergistically with miR-338-3p which represses the expression of FX to promote M2 macrophage polarization [86]. Deeper investigations of the probable role of Vitamin K in lncRNA expression regulation are warranted.

## Functional link between water-soluble vitamins (B AND C) and long non-coding RNAS

### Vitamin B group

The vitamin B group plays key roles in a wide range of cellular functions: metabolism, transport of nutrients, and synthesis of red blood cells [87]. There are eight types of vitamin B, each with unique functions: vitamin B1 (Thiamine), B2 (Riboflavin), B3 (Niacin), B5 (Pantothenic acid), B6 (Pyridoxal), B7 (Biotin), B9 (Folate), and B12 (Cobalamins). Each B vitamin is either a cofactor (generally a coenzyme) or a precursor of essential enzymes for several metabolic pathways as well as for RNA and DNA biosynthesis and DNA repair [87]. Mitochondrial dysfunction, neurocognitive disorders, and immune dysfunction are associated with B vitamins deficiency. In aging, B vitamins deficiency is also linked to osteoporosis, and cardiovascular disorders [88].

#### Vitamin B metabolism-associated disorders in prenatal and early childhood

Dietary deficiency of folate and B12 can lead to reduction of the insulin‐like growth factor type‐II receptor (IGF2R) levels in the placenta and the hepatic tissue of the fetus. Different dietary combinations of folic acid and B12 impact the epigenetic status of IGF2R and the lncRNA KCNQ1OT1 in placenta and fetus of C57BL/6 mice [89]. Folate-deficiency-induced changes in the expression of IGF2R were associated with enhancement of suppressive histone modifications. In addition, over-supplementation with either folate or B12 enhanced the expression of IGF2R and the lncRNA KCNQ1OT1 in the placenta and fetal tissues, and notably, up-regulation of KCNQ1OT1 exhibited a sex-biased pattern [89] (Table 4). Maternal vitamin B12, B6, and homocysteine levels impact offspring weight and DNA methylation at four differentially methylated regions (DMRs) involved in fetal growth and development during pregnancy [90]. These DMRs included the lncRNA maternally expressed gene 3 (Meg3), known for its functions in embryonic development [91], and H19, a lncRNA strongly expressed during embryogenesis [92]. Vitamins B12 and B6 were associated with 3-year weight gain. Moreover, maternal B6 concentrations were positively associated with methylation at the MEG3 DMR, highlighting how nutrients affect developmental epigenetics [90].

**Table 4:** Evidence of functional relationships between vitamin B and long noncoding RNAs (lncRNAs)

**Compound**	**LncRNA(s) studied in the project**	**System: Patients / cell line(s) / animal model**	**Highlighted lncRNA(s)/pathway(s)/target(s)/ partners/ interactions**	**Disease context/ implication for human disease**	**Main result(s):**	**Ref**
**Vitamin B metabolism-associated disorders in prenatal and early childhood**						
**Folic acid and B**_**12**_	KCNQ1OT1	C57BL/6 mouse model (Blood, serum [to assess biochemical parameters] Placenta and fetal tissues [to assess expression and epigenetics)	**_**	Effect of folic acid and B12 deficiency on offspring	The epigenetic reprograming of insulin‐like growth factor type‐II receptor (IGF2R) and lncRNA KCNQ1OT1 is associated with different dietary combinations of folic acid and B12 in mice	[89]
**Vitamin B groups**	H19, MEG3	496 infant-mother pairs	_	Effects of maternal vitamin B12, B6, and homocysteine consumption during pregnancy on fetal growth and development	• Association of maternal B12 and PLP concentration with 3-year weight gain was reported Association of maternal PLP with methylation status of MEG3 differentially methylated region was reported	[90]
**Vitamin B1 and B**_**12**_	MALAT1	Cerebral palsy model in rats Cell culture: Mouse N2A neuroblastoma cells	MALAT1/miR-1/BDNF/PI3K/Akt pathway	Cerebral Palsy (CP)	The protective effect of vitamin B1 and B12 on neuron injury is exerted through modulation of MALAT1/miR-1 axis	[93]
**Vitamin B group & folate metabolism**	RNA sequencing of Ang II exposed SH-SY5Y cells (432 upregulated & 389 downregulated lncRNA)	Ang II exposure in SH-SY5Y cells (human neuroblastoma cell line)	_	Ang II-related neuronal damage	Analysis of DE lncRNAs highlighted vitamin B group metabolism, glycosphingolipid biosynthesis, apoptosis, and the neurotrophin signalling pathway	[94]
**Vitamin B metabolism-associated disorders during adulthood**						
**Pyridoxal phosphate and pyridoxamine phosphate, folic acid, vitamin C**	Blood transcriptomic of TB patients vs controls (To find differentially expressed lncRNAs, mRNAs, and miRNAs)	Bioinformatics: (Computational analysis of public datasets)	OSBPL10-AS1/hsa-miR-485-5p/SLC23A2,	Pulmonary Tuberculosis (TB)	LncRNA OSBPL10-AS1, miRNA hsa-miR-485-5p, mRNA SLC23A2, pyridoxal phosphate, pyridoxamine phosphate, and folic acid are co-regulated blood biomarkers for TB	[95]
**Vitamin B**_**6**_	MALAT1	127 pairs of paraffin embedded breast IDC tissues and adjacent normal tissues, 105 benign fibroadenomas, MCF12A, MCF-7 and MDA-MB231	MALAT1/miR-216b-5p/PNPO axis	Breast Invasive Ductal Carcinoma (IDC)	The study identified a regulatory axis of MALAT1/miR-216b-5p/PNPO with potential to be therapeutically targeted	[96]
**Vitamin B**_**12**_	AspocR lncRNA	Listeria monocytogenes	_	L. monocytogenes and enterobacterial pathogenesis	Study showed that riboswitches modulate the expression of noncoding RNAs	[97]
**Vitamin B**_**6**_	Sense lncRNA AK370814	Seeds ((Hasat, Beysehir 99, Konevi 98 and Tarm 92) in response to salinity application for 3 days post-germination)	_	Germination under salinity condition	This lncRNA has been associated with vitamin B6 salvage pathway	[98]

Cerebral palsy is a group of disorders that appear in early childhood and affect movement and muscle tone [99]. Hypoxia-induced and ischemic brain damage is one of the main causes, while neuronal apoptosis is the main mechanism of nerve injury in cerebral ischemia [100]. Hydro-acupuncture (HA) injection of vitamin B1 and B12 in a cerebral palsy rat model ameliorated nerve injury, by affecting neuronal apoptosis via the MALAT1/miR-1/BDNF axis and the downstream PI3K/Akt pathway [93]. Vitamins B1 and B12 suppressed neuronal apoptosis by upregulating BDNF (brain-derived neurotrophic factor) [93], a promoter of neuronal survival [101]. Furthermore, Oxygen Glucose Deprivation/Reoxygenation (OGD/R) treatment in neurons induced apoptosis, repressed the expression of MALAT1 and BDNF as well as the phosphorylation of PI3K and Akt, and enhanced miR-1 expression. All these effects were reversed by vitamin B1 and B12 treatment [93] (Table 4). Intriguingly, BDNF is regulated by its endogenous *cis*-antisense lncRNA, BDNF-AS1 [102]. Hence, future work in this field should determine whether BDNF-AS1 is pertinent to the regulatory networks of the B vitamins. Finally, MALAT1 interference abrogated the neuroprotective action of vitamin B1 and B12. Taken together these results indicated that vitamin B1 and B12 specifically act on the MALAT1- miR-1 interface [93]. Also in the context of neuronal disorders, besides confirming the involvement of vitamin B metabolism in Ang II-related cognitive impairment, the target genes of certain differentially expressed lncRNAs contribute to vitamin B group (lipoic acid, folate, and vitamin B6) metabolism [94] (Table 4).

#### Vitamin B metabolism-associated disorders in adulthood

Patients affected with pulmonary tuberculosis (TB) exhibit abnormal concentrations of pyridoxal phosphate, pyridoxamine phosphate (two forms of vitamin B6), and folic acid [95]. This abnormal regulation of vitamin B metabolism may result from mRNA–lncRNA–miRNA network disruption due to the infection. Specifically, a ceRNA regulatory network consisting of 23 lncRNAs, 10 miRNAs, and 113 mRNAs participates in vitamin B metabolism regulation in TB patients. This integrated analysis also showed that lncRNA OSBPL10-AS1, miRNA hsa-miR-485-5p, and mRNA SLC23A2, along with the three vitamin B metabolites, constitute an integrative biomarker signature which reflects vitamin metabolism deregulation in TB patients, and may serve as promising blood biomarkers for an accurate diagnosis of TB [95]. The relationship vitamin B and the lncRNA MALAT1 is also correlated with the development of human breast invasive ductal carcinoma (IDC) [96]. Pyridoxine 5′-phosphate oxidase (PNPO), a converting enzyme for the active form of vitamin B6, pyridoxal 5′-phosphate (PLP), is overexpressed in human ovarian cancer, and PNPO suppression can inhibit proliferation, migration, invasion and colony formation of breast cancer cells [96]. Besides, PNPO positively correlated with MALAT1 in breast cancer cells, whereas MALAT1 was negatively correlated with miR-216b-5p, suggesting a ceRNAs regulatory mechanism [96]. Therefore, the MALAT1/miR-216b-5p/PNPO axis plays a key role in IDC development, and may have the potential to be therapeutically targeted [96] (Table 4). A riboswitch is a regulatory portion of a messenger RNA molecule, generally located in the 5' untranslated region, that binds a specific cognate small-molecule ligand and ultimately regulates the translation of the protein encoded by the mRNA [92]. Paradoxically, riboswitches may also regulate noncoding RNAs [97]. A vitamin B12-regulated riboswitch in *Listeria monocytogenes* regulates the expression of *AspocR*, a *cis*-encoded antisense RNA (asRNA) transcribed from the opposite strand of the *locus* encoding the transcription factor pocR. PocR activates the expression of *pdu* genes, implicated in propanediol catabolism, and vitamin B12 is a cofactor of enzymes involved in this catabolic process. Summarily, *PocR* and *pdu* genes are regulated by B12 in bacteria [97] (Table 4). Evidence of functional relationships between vitamin B and long noncoding lncRNAs is summarized in Table 4.

### Vitamin C

Vitamin C is a hydrophobic vitamin that most plants and animals synthesize through a four-enzyme pathway from D-glucose or D-galactose. However, due to the absence of the gene encoding a key enzyme in this biosynthetic pathway, gulonolactone oxidase, which is present in most non-primate mammals and beyond, humans need to obtain vitamin C from dietary intake. Vitamin C serves as an antioxidant to scavenge deleterious free radicals and enzyme cofactors for many reactions involved in the biosynthesis of collagen, carnitine, and neurotransmitters, coagulation factor V. It is absorbed through the intestine, transported into the blood by Na-dependent vitamin C transporters SVCT-1 and SVCT-2, and excreted unchanged through urine. Vitamin C can modulate gene expression [103].

Exogenous vitamin C has been shown to enhance proliferation, inhibit apoptosis, and reduce the global nucleic acid methylation levels of immature Sertoli cells. This type of treatment has resulted in differential expression of approximately 1000 lncRNAs with functions including oxidoreductase activity, cell proliferation and apoptosis, modulation of hormonal levels, modulation of catalytic activity, developmental processes, ATP metabolism, and reproductive processes [104]. Moreover, vitamin C has been found to exert anti-cancer effects in colorectal cancer (CRC) cells related to the MALAT1 lncRNA [105]. Vitamin C can suppress proliferation of CRC cells, induce apoptosis, and arrest cell cycle in the S phase, by downregulating MALAT1 [105]. Vitamin C treatment of donor cells may enhance cloned bovine embryo development through transcriptional regulation, including of lncRNAs [106]. Also, during the reprogramming of female somatic cells into induced pluripotent stem cells (iPSCs), vitamin C keeps the lncRNA X-inactive specific transcript (Xist) repressed, providing further evidence of the connection between vitamin C and crucial regulatory lncRNAs in stem cells and early development [107]. The link between vitamin C and lncRNAs is summarized in Table 5.

**Table 5:** Evidence of functional relationships between the Vitamin C group and long noncoding RNAs (lncRNAs)

**Vitamin**	**LncRNA(s) studied in the project**	**System: Patients / cell line(s) / animal model**	**Highlighted lncRNA(s)/pathway(s)/target(s)/ partners/ interactions**	**Disease context/ implication for human disease**	**Main result(s)**	**Ref**
**Vitamin C**	Differentially expressed mRNAs (1232) and lncRNAs (937) after treatment with ascorbic acid	Swine testicular cell line	MAPK, AMPK, PI3K-Akt and mTOR and pathways regulating cell proliferation and cell cycle	Reproduction of male animals	transcriptomic analysis demonstrated AA-induced differentially expressed mRNAs and lncRNAs enriched in MAPK, AMPK, PI3K-Akt and mTOR and pathways	[104]
	MALAT1	Human CRC cell lines: LS174T, HT-29, and SW480 Athymic nude mice (Balb/c, nu/nu)	_	Colorectal Cancer	Vitamin C could suppress proliferation, induce apoptosis and arrest cell cycle. More killing effects were observed in cells with high MALAT1 expression	[105]
	RNA sequencing of donner cells before and after vitamin C treatment/ differentially expressed mRNAs and lncRNAs	Oocytes and donor fibroblasts of bovine origin	_	improve cloned bovine embryo formation by somatic cell nuclear transfer from fibroblast donor cells	Vitamin C improved colony formation by restoring mRNA and lncRNA expression signature	[106]
	X-inactive specific transcript (Xist)	Mouse embryonic fibroblasts (MEFs)	_	Reprogramming of female somatic cells into induced pluripotent stem cells (iPSCs)	Vitamin C has a key role in keeping Xist repressed, reactivating the X chromosome during the pre‐iPSC to iPSC transition	[107]

Generally, although more data is needs to extrapolate through which lncRNA-dependent regulatory axes vitamin C might exert its role, molecular evidence preliminarily confirms the lncRNA-dependent protective roles of vitamin C.

### Vitamin metabolism and digestion

Evidence is progressively pointing toward a link between vitamin metabolism and lncRNAs (Table 6). For instance, the lncRNA-mRNA network constructed in gastric adenocarcinoma has shown the possible role of the AC115619.1-APOA4/APOB and AP006216.2-APOA1/APOA4 axes in the pathogenesis of this cancer through regulation of fat digestion and absorption as well as vitamin digestion and absorption [108].

**Table 6:** Evidence on specific functional relationships between vitamin metabolism pathways and long noncoding RNAs (lncRNAs)

**Compound**	**LncRNA(s) studied in the project**	**System: Patients / cell line(s) / animal model**	**Highlighted lncRNA(s)/pathway(s)/target(s)/ partners/ interactions**	**Disease context/ implication for human disease**	**Main result(s)**	**Ref**
Vitamin digestion and absorption (soluble and non-soluble)	To identify differentially expressed lncRNAs and mRNAs, 375 gastric adenocarcinoma and 32 adjacent non-tumor tissues were selected from TCGA	Bioinformatics analysis of mRNA and lncRNA expression profiles of 375 gastric adenocarcinoma and 32 adjacent non-tumor tissues	[AC115619.1-APOA4 /APOB] [AP006216.2-APOA1/APOA4] HOTAIR C20orf166-AS1, PGM5-AS1, HOXC-AS3, HOXC-AS2 AC012531.1	Gastric Cancer (GAC)	• HOXC-AS3 cis-regulated the homeobox transcription factor genes HOXC8, HOXC9, HOXC10, HOXC11, HOXC12 and HOXC13 • Six lncRNAs (HOTAIR, C20orf166-AS1, PGM5-AS1, HOXC-AS3, HOXC-AS2, and AC012531.1) showed excellent diagnostic value as biomarkers for GAC	[108]
Vitamin digestion and absorption	RNA sequencing of Hu sheep liver (with and without heat stress) inferred 520 DE mRNAs and 22 DE lncRNAs	Liver tissue of Hu Sheep	DE mRNAs showed enrichment in particular pathways including vitamin digestion and absorption	Animal health (productivity & reproductive efficiency)	Lnc_001782, because of its potential cis-regulatory effect, may positively regulate APOA4 and APOA5, two genes that are its genomic neighbors	[109]

RNA sequencing of hepatic cells of Hu sheep exposed to heat stress has shown differential expression of 520 mRNAs and 22 lncRNAs. Notably, the differentially expressed mRNAs have been associated with biological processes including vitamin digestion and absorption. LNC001782, as one of the differentially expressed lncRNAs, has been suggested to affect expression of APOA4 and APOA5, thus regulating liver function [109].

## Conclusions and future directions

Emerging evidence suggests that lncRNAs are important components of regulatory networks through which multiple key vitamins exert their roles. In contrast to protein-coding genes, lncRNAs are generally not well-conserved between closely-related species and lineages in evolution [2, 110, 111] and in particular, most human lncRNAs are not conserved beyond primates. Therefore, results obtained from non-primate animal models should be translated into clinical applications with caution. This necessitates design of appropriate experimental systems to define the physiological functions of lncRNAs, particularly human primary cell cultures, organoids, and nonhuman-primate models. This field can also benefit from human studies in people with rare genetic deficiencies in certain vitamins, to the extent that lncRNA functions underlie the corresponding phenotypes. High-throughput RNA sequencing assays before and after supplementation with these vitamins can help to identify the vitamins’ regulated genes globally in an unbiased fashion. Moreover, the association between vitamin deficiency and susceptibility to certain disorders can facilitate identification of the possible lncRNA/miRNA/mRNA targets of vitamins in each such disease.

Cancer, developmental and neuropsychiatric disorders were identified in our survey as “leitmotif” diseases recurrently connected with a few specific lncRNAs at the vitamin interface (see Tables 1 and 4). Among these lncRNAs, we spotted specific classical lncRNAs such as H19, MEG3, MALAT1, HOTAIR, and SNORNA host genes (SNHG6/16). They have been known in the field for decades and are its de facto “low-hanging fruit,” thanks to their clear associations with multiple diseases through diverse but well-understood mechanisms of action in the literature.

One of the major findings of this review is the frequent association of the H19 lncRNA with vitamin-driven regulation in disease, spanning disorders from Ankylosing Spondylitis to a range of cancers (Tables 2 and 4). Presumably this is due to the centrality of H19-driven downregulation of VDR, which is mechanistically well-characterized. Pharmaceutical industry should, perhaps, consider investing in developing an inhibitor of this downregulation, because such a drug might have broad relevance to restoring vitamin D function and treating the wide range of diseases whose etiology depends, at least in part, on H19-conferred vitamin D resistance.

MEG3, another classical lncRNAs that has been exhaustively studied in the past two decades,was mostly seen as associated with colorectal cancer across multiple vitamin-related studies. There is broad evidence, as reviewed here, from independent groups supporting the role of vitamin D-mediated pathways in the link between MEG3 and colorectal cancer.

MALAT1, one of the best-characterized lncRNAs and one of the most highly expressed genes in humans, was revealed by multiple groups to be relevant to breast cancer as well as to coronary artery disease, specifically as viewed through the prism of its interface with vitamin-mediated pathways. Summarily, this review places MEG3, MALAT1, and H19 at this interface as frequent, recurrently seen, and hence major lncRNA effectors of the vitamin-to-disease connection. However, our review also implies that unbiased whole-transcriptome studies of this connection, rather than studies centered on already-known lncRNAs, should be more frequent as they may identify additional lncRNAs uniquely relevant to vitamin-dependent disease etiology.

HOTAIR, previously known mainly for its oncogenic functions [112], is an lncRNA that more recently has been implicated in the pathogenesis of diseases other than cancer, including multiple sclerosis [47]. Surprisingly, our literature survey has uncovered evidence, from several groups, of HOTAIR’s relevance to multiple sclerosis pathogenesis linked to vitamin-dependent mechanisms, and hence adds to the body of knowledge about this lncRNA’s disease impacts, underscoring its protean and multifunctional versatility.

SnoRNA host genes are notable because snoRNAs essential to ribosome component biosynthesis in the nucleolus may also act through poorly understood systemic noncanonical pathways [113]. Here we establish broad relevance of 2 snoRNA host genes, SNHG16 and SNHG6, to a variety of cancers and neuropsychiatric disorders specifically through their relationship with vitamins, well supported by multiple studies (Table 2). The strength of this connection clearly indicates that the role of snoRNA host genes and snoRNA biogenesis and ribosomal as well as noncanonical functions should all be subject to functional investigation within the specific context of vitamin D-driven regulation, given that vitamin D is the sole vitamin associated with a bevy of SNHG6/16-linked disease outcomes in humans and animal models. The role of snoRNA in cancer is well-established [114]. Interestingly, there are practically no publications to date examining snoRNA host genes in neurological and neuropsychiatric diseases. Hence it is possible that our vitamin-centric review approach has uncovered a novel set of snoRNA host gene action modalities in those groups of diseases that, in contrast to the role of snoRNA in cancers, have not yet been subject of much functional investigation.

Prior to our review, the firm relevance of these lncRNAs that we have listed above—as a group of “famous, by-now-classical, lncRNAs” – to vitamin pathways was not explicitly, and integratively, summarized across multiple studies. This review should motivate RNA biologists to even deeper investigate the already-well-known molecular mechanisms of these lncRNAs in order to understand how they serve as the regulators and/or the targets of the vitamins that we discussed.

Our search revealed that vitamin D is the best-studied vitamin showing association with lncRNA in various disease contexts. In particular, the lncRNA-mediated beneficial effect of vitamin D in treating neuropsychiatric disorders was evident. The positive effect of vitamin D supplementation for treatment in psychiatric illness is well-supported too [115, 116]. The demonstrated regulatory axes in those studies provide well-defined molecular evidence for the potential of a synergistic effect between vitamin D and the drugs commonly prescribed for these disorders. One implication for clinicians might be to consider prescribing vitamin D supplementation in the early stages of disease, or for neuropsychiatric disorders with a clearly defined genetic component for family members at risk. 1,25(OH)_2_D_3_ can induce the differentiation of acute myeloid leukemia cell lines to more mature monocytic cells [117] Hence, further research to consider the effectiveness of co-use of existing drugs and vitamin D derivatives in lncRNA-dependent pathways defined in a particular disease context could be a worthwhile area of research. Because the lncRNAs in these networks may serve as both biomarkers and drug targets, more effective “theranostic” approaches might be envisioned.

Vitamin D can act through both canonical and nonclassical pathways with its faithful classical partner VDR, or non-VDR dependent pathways (Fig. 3) [118]. On the other hand, VDR may have an anti-apoptotic function independent of 1,25(OH)_2_D_3_ [119]. Comprehensive insights about different modes of vitamin D actions are summarized in an in-depth review [118]. The lncRNA H19 has opposite functions in different vitamin D-regulated contexts, perhaps because of its involvement in VDR-dependent as well as noncanonical pathways. Two H19-dependent regulatory axes together affect the expression of VDR and increase the inflammatory response in Ankylosing Spondylitis (AS). The H19-miR675-5p-VDR pathway increases VDR expression level in AS, but in ulcerative colitis, H19 overexpression decreased VDR expression through the same pathway [120]. One possible explanation might be related to the non-genomic actions of vitamin D / VDR that obscure the extent to which a particular regulatory axis might contribute to the final output in various disease contexts vitamin D affects the inflammatory response through nonclassical pathways. Future studies should dissect both the classical and non-classical roles of vitamin D / VDR to avoid such research bias.

One of the applied challenges, especially for the fat-soluble vitamins, is defining adequate dosing for optimal health while avoiding side effects. Rational therapy design based on molecular understanding of regulatory pathways may pave the way for individualized prescriptions and doses of vitamins according to the individual patients’ circulating biomarker lncRNA expression profiles. The advent of such individualized prescriptions in the pharmaceutical market would help to capture the post-genomic promise of true precision medicine based on patient-specific transcriptome quantification. Mechanistically, several lncRNAs that participate in regulatory networks with vitamins may serve as molecular sponges for miRNAs, consistent with the ceRNA model. Therefore, deciphering the functional network connecting lncRNAs and miRNAs may help in understanding the mechanisms of lncRNA-mediated regulation of vitamin-related signaling pathways. Recent studies have highlighted the impact of vitamin-related signaling pathways in a wide array of human disorders, including cancers, neuropsychiatric conditions, and congenital malformations. Therefore, lncRNAs directly contributing to the etiology of these disorders through known pathways should be considered as putative therapeutic targets. Further functional investigations in this field will further elucidate the molecular mechanisms at play and hence are expected to facilitate the design of novel lncRNA-based therapies for these disorders.
