# Correction: Tracing vitamins on the long non-coding lane of the transcriptome: vitamin regulation of LncRNAs

**DOI:** 10.1186/s12263-024-00746-5

**Published:** 2024-06-18

**Authors:** Fatemeh Yazarlou, Fatemeh Alizadeh, Leonard Lipovich, Roberta Giordo, Soudeh Ghafouri-Fard

**Affiliations:** 1 Center for Childhood Cancer, Abigail Wexner Research Institute at Nationwide Children’s Hospital, Columbus, OH USA; 2 College of Medicine, Mohammed Bin Rashid University of Medicine and Health Sciences, Box 505055, Dubai, United Arab Emirates; 3 Department of Genomic Psychiatry and Behavioral Genomics (DGPBG), Roozbeh Hospital, School of Medicine, Tehran University of Medical Sciences, Tehran, Iran; 4 Department of Biology, College of Science, Mathematics, and Technology, Wenzhou-Kean University, Wenzhou, Zhejiang Province China; 5 Shenzhen Huayuan Biological Science Research Institute, Shenzhen Huayuan Biotechnology Co. Ltd., 601 Building C1, Guangming Science Park, Fenghuang Street, Shenzhen, Guangdong 518000 China; 6 Center for Molecular Medicine and Genetics, School of Medicine, Wayne State University, 3222 Scott Hall, 540 E. Canfield St., Detroit, MI 48201 USA; 7 Department of Biomedical Sciences, University of Sassari, Viale San Pietro, Sassari, 07100 Italy; 8 Department of Medical Genetics, Shahid Beheshti University of Medical Sciences, Tehran, Iran

**Correction to: Genes & Nutrition**

10.1186/s12263-024-00739-4

Following publication of the original article, it was brought to the journal’s attention that the affiliations information of the article was incomplete. Namely, ‘Center for Childhood Cancer’ was missing from affiliation 1, while the following affiliation was missing for Leonard Lipovich: Department of Biology, College of Science, Mathematics, and Technology, Wenzhou-Kean University, Wenzhou, Zhejiang Province, China.

The affiliations information has since been corrected in the published article and the corrected affiliations information may be seen in this erratum. The authors thank you for reading and apologize for any inconvenience caused.

